# Does User Preference Matter? A Comparative Study on Influencing Factors of User Activity Between Government-Provided and Business-Provided Apps

**DOI:** 10.3389/fpsyg.2022.914528

**Published:** 2022-06-23

**Authors:** Yuanyuan Guo

**Affiliations:** Department of Government Management, Beijing Normal University, Beijing, China

**Keywords:** government-provided app, business-provided app, digital government, user behavior, user preference

## Abstract

The competition between government-provided apps and business-provided apps for active users in China is becoming increasingly fierce. Apps with higher user activity will win this competition. To maintain active users, finding the user activity influencing factors is crucial. In this study, we selected a government-provided app—“Beijing One Card”- and a business-provided app—“Bus Code” (the Beijing area) -in the field of public transportation as the comparative research objects. Based on multiple regression analysis, we explored the factors influencing user activity. We found user preference plays a critical role in distinguishing the influencing factors of user activity between two kinds of apps. Distinct from the existing research, the service quality does not affect the user activity of public transportation apps. This paper argues that, whether it is a government-provided app or a business-provided app, to enhance the user activity of the app, in the short term, it is necessary to improve the app function according to the user’s needs, try to provide services which user like, cater to user’s preference, strengthen user experience; In the long run, it is essential to pay attention to mining the user’s data accumulated while operating the app to understand user behavior. So as to affect the user’s preference, enhance user’s trust, and improve user participation through public policy.

## Introduction

At present, the Chinese government combines the reform idea of “Internet + government service” to provide public services through apps, websites, We-chat, and other ways in various fields, including public transportation. However, with the endless stream of apps provided by the government, some apps lack active users or have low user activity. The government has invested a lot of money in government apps, but the benefits are minimal. Users rarely use or are unwilling to use government apps. In addition to the apps provided by the government, enterprises are also providing apps with similar service functions to compete for active users. Taking the public transportation apps in Beijing as an example, there are many public transportation apps, such as “Bus Code,” “Easy Access,” “Beijing One Card,” “Beijing Bus,” and “Beijing Subway.” Some apps’ users are very active. This kind of app always remains a high download amount on the app download rank. However, some apps are gradually becoming “neglected” apps, losing active users, and few people download and use them. This phenomenon leads to myriad problems, such as: “what are the influencing factors of user activity in government-provided and business-provided apps?” “what are the differences between these two kinds of apps’ influencing factors?” and “what should governments and enterprises do to improve apps’ user activity?”

In the field of public administration, scholars mainly discuss these research propositions from two dimensions: willingness to use continuously (Chen et al., 2017; Li et al., 2019; Guo and Ming, 2020; Jiang and Lv, 2020; Liu and Wei, 2020; Vinerean et al., 2022) and behavior to use continuously (Chen Q. et al., 2020; Ming and Cao, 2020; Zhang et al., 2020). They took these two dimensions from the citizen’s perspective as the research subject (Chen et al., 2017; Chen Q. et al., 2020; Li et al., 2019; Guo and Ming, 2020; Jiang and Lv, 2020; Liu and Wei, 2020; Ming and Cao, 2020; Zhang et al., 2020). However, the service provider (government/enterprise) and the promotion process are also valuable research subjects. If we combine these three dimensions and consider them as one research subject, the research will be more thorough. It is worth noting that citizens also have the attribute of consumers. They can use public products provided by the government as citizens and use commercial products provided by enterprises as customers. Combining with the D&M IS success model (DeLone and McLean Information System success model), technology acceptance model, and 4Ps theory, this study integrates the service provider (government/enterprise), the service demander (citizen), and the promotion process to study the influencing factors and differences of various apps’ user activity, which makes up for the deficiency of related research. In this study, we selected the “Beijing One Card” app provided by the government (a government-provided app) and the “Bus Code” app (the Beijing area) provided by the enterprise (a business-provided app) as the comparative objects. They both have the highest user usage in the pre-survey of the Beijing area. Through multiple regression analysis, we explored the main factors affecting the user activity of these two apps in terms of users’ subjective experiences and use feelings. We also summarized the improvement suggestions for government-provided apps and business-provided apps, which play a vital role in further improving the user activity and retaining active users of government/business-provided apps.

## Literature Review and Research Hypothesis

### Literature Review of the Government Apps

The research on government apps has two types: One is the research on the function of the government apps themselves. The other is the research on users who use government apps.

Given the problems of government apps themselves, scholars mainly discuss the research of interface design, system programs, and so on. For example, Xue and Xie (2015) believe that the problems in the development and operation of government apps in the early stage including the overall chaos, different standards, blind development. Besides, most government apps’ function setting is not perfect, and it is useless to overlap with the existing application functions. The content operation is not active too. There are many zombie applications, which means a small number of users with poor viscosity. He and Zhang (2020) believe that government apps combine the advantages of government microblog, WeChat convenience, and government website aggregation, which are vital tools to promote “Internet + government service.” Taking Changsha City as an example, they investigated and analyzed the construction status of local government apps from the five directions of the government: user, app architecture, and the dimensions of content update, office efficiency, and interactive response. They found blind development, severe formalism, and poor safety performance will cause apps to become “ignored apps.” Under the background of “Internet + government service,” the local government app needs to make full efforts from the aspects of construction, application, and management, innovate government service mode and improve government service level. Xu and Hua (2020) constructed an evaluation index system of a government affairs app, including five first-level indicators, such as information service, transaction service, and service participation, and 22 second-level indicators, such as information usefulness and practical and reliable information sources. Considering the heterogeneity of expert groups, the diversity of decision-making information, and the fuzziness of expert judgment in the evaluation process, they established the information consistency processing method. Then, they introduced the intuitionistic fuzzy analytic hierarchy process to determine the index weight and evaluation results. Finally, they took five representative government affairs apps such as “Zheliban” as examples for empirical research. By analyzing the evaluation results, they put countermeasures and suggestions for improving the government app from the construction mode, system design, and functional innovation.

Research on users using government apps including the research of user perception (Zhao and Yang, 2015; Meng and Zhu, 2017; Sun et al., 2017; Simarmata et al., 2019), user satisfaction (Chen et al., 2017; Wang and Ding, 2019; Pan, 2020), user willingness to use (Li et al., 2019; Wang and Ding, 2019; Zhang et al., 2019; Liu and Wei, 2020), user continued to use (Chen et al., 2017; Ming and Cao, 2020; Zhang et al., 2020), and so on. Wang and Ding (2019) studied user satisfaction and users’ willingness to continue to use. Based on the technology acceptance model, the information system success model, and the information system sustainable use model, they constructed the influencing factor model of the public willingness to continue to use government service apps. They found that user satisfaction, perceived usefulness, perceived ease of use, system quality, degree of expectation confirmation, and other factors have a significant indigenous impact on the public willingness to continue using government service apps. They suggested that the government should further strengthen the publicity of government service apps, pay more attention to optimizing and improving the design and function of apps, and continuously improve public satisfaction and willingness to use apps. Zhao and Yang (2015) studied the issue of user adoption. Based on the existing literature and models, they selected factors such as interactivity, mobility, situational awareness, critical quality, perceived ease of use, perceived usefulness, and perceived encouragement to construct the model of public adoption of government apps. From the perspective of users’ willingness to use, Zhang et al. (2019), based on the S-O-R theory, constructed a model of influencing factors of mobile government app users’ willingness to use from two aspects of external stimulation and internal transformation, and designed a questionnaire and conducted empirical research accordingly. They found that perceived usefulness, relative advantage, and satisfaction directly and significantly impact the willingness of mobile government app users. Information quality, system quality, and service quality will indirectly affect the willingness of mobile government app users.

The existing research on government apps shows that, because the construction of government apps is the “pillar” of China’s information construction, China urgently needs high-level research results. However, the current research on government apps is challenging to meet practice needs. This phenomenon can be summarized as “several aspects more, and several aspects lack.” Specifically, in the relevant research of government apps, there are more articles on “app” and “user,” and fewer articles on ‘the provider’-government or enterprise. Too much articles focused on academics and policy research and a few articles focused on business application. There are many studies on local problems and few articles on the overall situation of the system. There is much research focusing on the continuous use of users, but there is little research on the application of user preference and user psychological selection mechanisms. It is worth noting that scholars have paid attention to domestic research in China without considering the introduction of foreign theories, policies, and practices. Furthermore, at the policy level, the progress at the policy level still lacks sufficient support at the theoretical level. Theoretical research lags behind policy formulation needs, and the combination of qualitative and quantitative research is lacking. Finally, judging from the academic achievements published by government apps at the present stage, most of them are the results of investigation and research on some aspects of the development of government apps. There are many articles and reports on emergencies and scenarios, but systematic research with theoretical depth and empirical support is rare.

### Literature Review of Public Transportation Apps

At present, there are three main aspects of research on a public transport app.

Firstly, the research on influencing factors includes user satisfaction, user loyalty, user continuance intention, and user interest. Chan et al. (2020) believe that the determinants of user satisfaction and loyalty to public transport apps include accessibility, reliability, comfort, and safety. If real-time global positioning system tracking is integrated into the public transport app, it will improve users’ satisfaction and loyalty to the public transport app. In addition, they suggest that future research may combine service quality, usability, and perceived value in the study of user satisfaction and loyalty. Simarmata et al. (2019) examined the user interest in commercially available public transportation apps and found that the app’s image, ease of use, price, and promotion significantly influenced user interest.

Secondly, the research about different user groups, including the elderly, youth, and other groups (disabled people, children, etc.). Koc and Kaya (2021) believe that responsiveness, politeness, punctuality, information accessibility, tangibility, occupancy, and security affect the use of public transport apps by students, staff, and academics on university campuses. Kim and Sik (2020) examined whether the information design of public transportation apps is appropriate for older people and provided guidance on information design for older people. They point out that Busan’s public transport information design conveys a lot of the information supplied by smartphone applications. However, the application’s availability is limited for the elderly who are not familiar with the smartphone. Therefore, they proposed information design criteria based on readability, visibility, and operability.

Finally, public transportation and its apps are also related to smart city planning. Some articles (Konecny et al., 2021; Lãzãroiu and Harrison, 2021; Nica, 2021; Woodward and Kliestik, 2021) have focused on the relationship between urban big data analytics and sensing and computing technologies for public transportation apps. Besides, some articles (Adams et al., 2021; Campbell et al., 2021; Wallace and Lãzãroiu, 2021) also focused on the relationship between predictive control algorithms and sensor-based big data applications regarding public transportation apps. For example, Wallace and Lãzãroiu (2021) analyze and evaluate the factors that influence people’s intention to use self-driving cars from three aspects: road abnormality detection, motion planning, and tracking control algorithm.

It can be seen from the above literature that in the existing research on public transport apps, there are mainly the following deficiencies. First, there is no distinction between providers. That is, there is no distinction between public transport apps provided by the government or provided by enterprises. When a user chooses a public transport app, he or she may select his or her favorite provider. Second, there is less targeted research on the public transport app provided by the government, let alone a comparative study between the two providers. The existing research is more unilateral research on enterprises’ commercial goals, and public transport apps. Apparently, user policies that are appropriate for business apps are not always appropriate for government apps. Third, there is a lack of overall attention to user activity research. Now, more and more public transportation apps are showing face, grab active users is the key to making app ‘survival’. It is crucial to understand whether the government-provided apps’ users are more active or the business-provided apps’ users are more active. Besides, the influencing factors of the user activity will provide valuable suggestions for the government and enterprises in the field of public transportation. For the government, understanding the influencing factors of user activity can enable the government to provide better digital services, improve user trust, maintain user activity, and improve citizen participation. For enterprises, understanding the influencing factors of user activity can maximize the enterprises’ interests while enhancing the user travel experience.

### Basic Theories

Scholars often use theoretical models, such as the D&M IS success model (Chen et al., 2016; Sun et al., 2017), the technology acceptance model (Du, 2010; Chen et al., 2016), etc., when studying users’ acceptance, continuous use, and activity. These theoretical models are well explanatory and widely praised by scholars (Du, 2010; Chen et al., 2016; Sun et al., 2017) who focus on public administration. This paper holds that combining these two common models can study users’ continuous use and activity of specific apps from a deeper perspective. In addition, introducing the 4Ps theory into the integration model can more clearly sort out the supply-demand relationship between the service provider (government/enterprise) and the service demander (citizen). Therefore, the theoretical basis of this study is to combine the D&M IS success model and the technology acceptance model with the 4Ps theory and add new variables.

#### DeLone and McLean Information System Success Model

In terms of the effectiveness of information systems or the success of information systems, DeLone and McLean proposed a widely accepted information system success model in 1992 (DeLone and McLean, 1992). An updated IS success model was then proposed, including six interrelated dimensions of IS success: information, system and service quality, (intention) use, user satisfaction, and net benefit. The model can be interpreted as: the system can be evaluated according to information, system, and service quality, which affect the subsequent use or intention and user satisfaction. The results of using the system will yield some benefits. Net benefits will (positive or negative) affect user satisfaction and the further use of information systems. They regard system quality, information quality, and service quality as independent variables; use intention and user satisfaction as intermediary variables; and net benefit as the dependent variable.

Some scholars apply this model to the study of user satisfaction (Liu et al., 2017), user continuance (Sun et al., 2017), and user adoption (Zhao and Yang, 2015; Chen et al., 2016; Sun et al., 2017; Karen et al., 2019; Huang et al., 2020). Chen et al. (2016) constructed the whole process e-government adoption model based on the D&M IS success model, combining the influencing factors and paths of e-government citizen adoption, and revealed the social benefits of citizen adoption of e-government. Huang et al. (2020), based on the D&M model, discussed the influencing factors of users’ willingness to adopt the think tank WeChat official account, provided inspiration, thought of improving the spread scope of the think tank WeChat official account, and enhanced the influence of think tanks. They found that information quality significantly affects performance expectation, service quality significantly affects effort expectation, and both of them affect perceived advantage, and affect users’ adoption intention through performance expectation, effort expectation, and perceived advantage. Liu et al. (2017) preliminarily constructed and verified the revised D&M model by comparing readers’ perception differences in mobile library system quality, information quality, and technology matching. They found that system quality, information quality, and service quality have positive effects on readers’ satisfaction and personal performance.

#### Technology Acceptance Model

Davis proposed the Technology Acceptance Model in 1989 (Fred, 1989) when he studied users’ acceptance of information systems based on rational behavior theory. The original purpose of this model is to explain the decisive factors of widespread computer acceptance. The technology acceptance model puts forward two main determinants: perceived usefulness, which reflects the degree to which a person believes in using a specific system to improve his or her work performance; perceived ease of use reflects the degree to which one thinks it is easy to use a particular system. The technology acceptance model shows that system use is determined by behavioral intention, and behavioral intention is determined by attitude toward the use and perceived usefulness. Attitude to use is determined by perceived usefulness and ease of use, perceived usefulness is determined by perceived ease of use, and internal variables determine external variables. External variables include system design characteristics, user characteristics (including perception form and other personality characteristics), task characteristics, the nature of the development or implementation process, policy influence, organizational structure, etc.

This theory is also used in user satisfaction, user continuous use, user adoption, user acceptance, and other research. For example, Du (2010), based on the technology acceptance model TAM, through the analysis of the two aspects of useful cognition and easy cognition, concluded that the main factors affecting the public acceptance of e-government are: the convenience of service access channels, the operability of the system, the one-stop level of e-government services, and the public’s awareness of e-government. The government must increase investment in public network infrastructure, increase the construction of a public service management system, understand the needs of the public, provide public services needed by the people, and actively promote e-government publicity.

#### 4Ps Theory

4Ps theory is developed from 4P theory. Philip (1967) further confirmed the marketing mix method with 4Ps as the core, namely, product, focusing on the function of development, requiring products to have unique selling points, and putting the functional demands of products first; price: the businesses according to different market positioning, formulate different prices, product pricing is based on the enterprise brand strategy, and pay attention to the brand value; place: enterprises do not directly face consumers but pay attention to the cultivation of distributors and the establishment of a sales network. The connection between enterprises and consumers is carried out through distributors; promotion: enterprises pay attention to the change of sales behavior to stimulate consumers, with short-term behavior to promote consumption growth, attract other brands of consumers or lead to early consumption to encourage sales growth; strategy: a series of strategies for customers.

### Theoretical Framework and Research Hypothesis

Whether it is a government-provided app or a business-provided app, its essence is service. The app is merely the platform to provide service. User activity of an app essentially reflects users’ continuous use decision-making behavior to meet their own consumption needs. Previous studies have mainly studied the factors that influence users’ decision-making on sustainable use of two dimensions: willingness to use continuously and behavior to use continuously.

The willingness to use continuously mainly focuses on the psychological feeling, and experiences of users, and users subjectively want to use the app. For example, Jiang and Lv (2020), based on the theoretical model of continuous use of information systems and introduced the relevant variables of experience value theory, constructed a user continuous use model of the AudioBook app with seven dimensions, including expectation confirmation, reading usefulness, social usefulness, auditory experience value, satisfaction, perceived entertainment, and return on investment value. They found that expectation confirmation positively affects reading usefulness, social usefulness, and perceived entertainment. Perceived entertainment, return on investment value, and auditory experience value positively affects user satisfaction. Reading usefulness, perceived entertainment, return on investment value, and satisfaction positively affect users’ willingness to use continuously. Liu and Wei (2020) put forward a model of users’ willingness to use continuously, which includes seven variables: useful perception, easy to use perception, enjoyment perception, expectation confirmation perception, convenience condition, community influence, and task technology adaptation. They also point out that trust perception, interactive perception, and quality level do not influence the willingness to use continuously. Zhang et al. (2020) think that personal information factors (individual characteristics, user habits, user motivation), information technology factors and information environmental factors are independent variables that affect users’ willingness to use continuously. Vinerean et al. (2022) pointed out that hedonic motivation is the key factor in consumers’ intention to continue using mobile commerce. Besides, social influence, performance expectancy, and trust also impact consumers’ continuous use intention.

The behavior to use continuously mainly concentrates on users’ use behavior, including users’ continuous use of an app, active time and frequency, etc. Chen M. et al. (2020) used the stimulus-organism-response model for reference, took technical factors (mobility, service quality) and social factors (subjective norms, trust) as stimuli, and user fit as moderating variables to build a model of continuous use behavior of government WeChat users. They found that mobility positively affects useful sense and easy-to-use sense; Service quality positively affects usability perception. Subjective norms and trust are two social factors that positively affect useful awareness and easy-to-use awareness. Useful awareness and easy-to-use awareness have positive effects on persistent use behavior, and user fit has a negative moderating effect on the relationship between easy-to-use sense and continuous use behavior. Ming and Cao (2020) took a different approach and extracted the influencing factors of users’ non-sustainable use behavior through grounded theoretical methods. They included 17 factors in three layers: user layer (unconfirmed expectations, social circle influence, unaccustomed, uncertainty, low self-efficacy), human-system interaction layer (low perceived ease of use, lack of personalization, high use cost, high perceived risk, high learning cost, low service quality), and system layer (attractive alternative products, lack of information resources, slow response, unreasonable navigation settings, unscientific privacy settings, imperfect functions).

Whether it is psychological users’ willingness to use continuously or actual users’ behavior to use continuously, they all impact users’ app active time and use frequency. Therefore, referring to the related literature on users’ willingness to use continuously and users’ behavior to use continuously, this paper puts forward the dependent variable—user activity. There is little research on user activity, but it provides research ideas for this paper. Zhou and Li (2011) argue that user activity is a measure of the users’ activity frequency of a website, and it is the product of user login frequency and login time. Besides, there are few comparative studies between government and business platforms.

According to the above literature analysis and based on the D&M IS success model and the technology acceptance model, the independent variables of user activity can be summarized into three aspects: taking the app as a subject, we select system quality (Wang and Ding, 2019), and service quality from the D&M IS success model (Li et al., 2019) as part of the independent variables; taking the publicity and promotion as the research object, combined with the research of existing literature, we developed the D&M IS success model’s information quality into promotion effect, including browsing (Zhou and Li, 2011) and comments; combining the attitude and behavioral intention in the technology acceptance model, we developed a new dimension: user preference. This dimension includes trust, interest, and habit. Combining the 4Ps theory in marketing (Philip, 1967), and from the perspective of both supply and demand, this paper regards the governments and enterprises as suppliers of products, users as demanders, and price, place, and strategy as the means in the process of promoting products (app). In this way, we can investigate the influence of service quality, system quality, promotion effect, and user preference on user activity.

**FIGURE 1:**
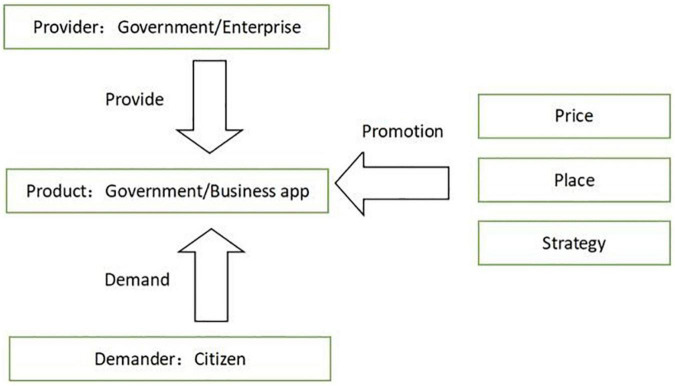
Basic model of apps’ user activity from the perspective of supply and demand sides.

Based on the above analysis, the model of influencing factors on user activity of government/business-provided apps proposed in this paper is shown in Figure 1.

#### Service Quality

Karen et al. (2019) pointed out that, when studying service quality, it is necessary to take users as the research subject and consider users’ perception of service through users’ observation. But Taylor and Baker (1994) think that service quality should have three dimensions: product, delivery, and environment. Joan (2003) evaluates e-government services based on the availability theory of e-services and puts forward a service quality model, consisting of three crucial dimensions: paying attention to users, user satisfaction, and result orientation. His model proposed is general, so any e-government service in this model applies.

Chen Q. et al. (2020) pointed out that service quality positively affects users’ sense of usefulness, which positively affects users’ continuous use behavior. The research of Siriluck (2008) also focuses on the risk perception model of e-government service quality, which consists of two main dimensions: e-government service quality and perceived risk. Research shows that the perceived value of e-government service consists of “service design, website design, technical support, and customer support.” Ming and Cao (2020) found that low service quality is the critical factor for users not to use continuously. Li et al. (2019) and others believe that service quality affects user satisfaction, and user satisfaction affects users’ continuous use. Sun et al. (2017) pointed out that service quality has a positive and significant impact on user satisfaction of library WeChat official account, while user satisfaction has a positive and significant effect on users’ willingness to continue using. Liu et al. (2017) constructed a basic model based on the successful integration model of D&M and TTF to compare readers’ perception differences in mobile library system quality, information quality, and technology matching and put forward theoretical assumptions. They found that the service quality positively impacts readers’ satisfaction and personal performance. Moreover, after readers get higher satisfaction, they will improve the degree of continuous use and sharing of the mobile library, thus showing higher activity. Huang et al. (2020), based on the UTAUT model and the D&M model, constructed the conceptual model of the WeChat official account’s adoption intention of think tank WeChat and discussed the influencing factors of users’ adoption intention of the think tank WeChat official account. They found that the service quality significantly affects the effort expectation, and the service quality impacts perceived advantage. In addition, service quality also affects users’ adoption intention through performance expectation, effort expectation, and perceived advantage. Chen et al. (2017) pointed out that information service quality and service quality significantly positively impact citizens’ intention of continuous use, reflecting users’ activeness. Combined with the above research ideas, this paper makes the following assumption:

Hypothesis 1: Service quality (SQ) has a significant positive impact on user activity.

#### System Quality

The system quality has a positive impact on user satisfaction (Liu et al., 2017). After users get higher satisfaction, they will increase the frequency of using the APP continuously, thus increasing the user activity. Based on the research on the technology acceptance model and the information system success model, Wang and Ding (2019) found that system quality has positively impacted the public’s willingness to use continuously. Pan (2020) points out system quality is the key factor affecting users’ willingness to use continuously by studying the online learning platform quality. Guo and Ming (2020) dynamically tracked and obtained data on users’ willingness to continue using and found that the influencing factors of users’ willingness to continue using are different, with different time nodes. At the initial stage, the system quality does not influence users’ willingness to use it continuously. However, after 3 months, it has an impact on users’ willingness to use continuously. Liu et al. (2017) believe that system quality has a positive impact on reader satisfaction and personal performance. Good system quality is the premise for readers to use the mobile library.

Meng and Zhu (2017) studied the quality of digital libraries through exploratory factor analysis, confirmatory factor analysis, and structural equation models to enhance user perception and increase user stickiness. They found that system quality positively impacts information quality, service quality, and perceived usefulness. Sun et al. (2017), based on the information system success model, from the perspective of user perception, discussed the influencing factors of the continuous use intention of library WeChat official account and found that system quality positively impacts user satisfaction. User satisfaction has a positive impact on users’ continued use intention. Ali et al. (2022) found that system quality is positively related to users’ intention to use mobile applications, that is, service quality affects users’ actual use of mobile applications. Their research confirms the positive impact of mobile applications on customer service and customer satisfaction. According to the above ideas, this study also makes the following assumption:

Hypothesis 2: System quality (XQ) has a significant positive impact on user activity.

#### Promotion Effect

The promotion effect of the platform (app) is the intermediate process connecting the government/business-provided apps with users. Feng et al. (2019), starting research on social capital, social trust, and user habit, pointed out that, compared with their behavior habits, the social trust formed by the public on the platform of government affairs Weibo has a greater impact on their communication-related government affairs Weibo behavior. Some scholars take the popularization results as the research aspect. Chen Q. et al. (2020) pointed out that videos of technology, music, and fashion themes can improve the overall communication effect, and technology videos have the highest contribution. Sitcom, real shot video, and surveillance video all positively and significantly influence the overall communication effect, and real shot video has the highest contribution. Video clip rate, vertical screen video, subtitle addition, and cooperative creation can significantly improve the overall communication effect. The vertical screen has the most outstanding contribution to the comprehensive communication effect model. Video duration and cover picture have no significant impact. Lin (2018) thinks that information disseminators (subject image, serviceability, service consciousness), the WeChat media platform (information utility, content quality, push frequency), noise interference (group interference, interest interference, publicity interference, competition interference), and information recipients (reading quantity, attention quantity, praise quantity, and forwarding quantity) impact promotion effect. Ali et al. (2022) found that system quality is positively related to users’ intention to use mobile applications, that is, service quality affects users’ actual use of mobile applications. Their research confirms the positive impact of mobile applications on customer service and customer satisfaction. According to these pieces of research, this paper makes the following assumption:

Hypothesis 3: The promotion effect (PE) of the platform has a significant positive impact on user activity.

#### User Preference

User preference is a vast concept that includes users’ trust, interest, and habits. User preference results from a total balance between cognition, psychological feeling, and rational economic trade-off. It is a sensible and tendentious choice made by users when choosing goods and services. Specifically, users often take actions under the control of the subconscious, and they will have special trust and interest in specific goods and services and repeatedly and habitually use certain kinds of goods and services. This kind of repetition and habit constitutes the user preference. Some scholars think the object factor is the primary factor in studying trust. Bai et al. (2014) analyzed the influencing factors of online transaction trust through the inter-group factor experiment method. They thought that website information type, brand, and service guarantee were the essential factors. Some scholars believe environmental factors are the key factors. Ma (2016) argued that users’ use of e-government would affect users’ trust in different aspects. Users’ perception of government transparency and response will mediate their correlation. In public administration, few pieces of research concentrate on influencing factors of interest; most of them concentrate on modeling and behavior prediction of users’ interests. Users usually collect their favorite pictures on social media platforms and label them with classification labels. Zhu et al. (2020) constructed a user interest graph model, representing a hierarchical tree structure, with an exponential interest attenuation mechanism. Their research in 2020 commented on this model from four dimensions: data collection, representation of user interest, construction and enhancement of user interest characteristics, and evaluation of the user interest model. Zhong (2016) and others also believe that habits positively and significantly impact user activity. Combined with previous research ideas, this study makes the following assumption:

Hypothesis 4: User preference (UP) has a significant positive impact on user activity.

**FIGURE 2:**
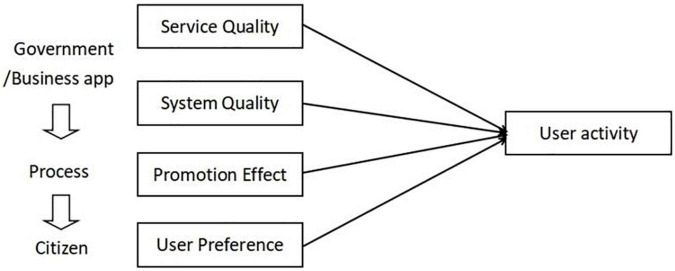
The basic influencing factors model of user activity.

Because this research is basic research and the proposed model (Figure 2) is still the basic model, the control variables have not been added yet. According to the principle of the multiple regression model, the expected formula of this study is as follow:


(1) $$Y=X_{0}+X_{1}^{*}SQ+X_{2}^{*}XQ+X_{3}^{*}PE+X_{4}^{*}UP+\varepsilon$$

X_1_, X_2_, X_3_, and X_4_ are coefficients of multiple regression equation, X_0_ is the constant and ε is the error term.

## Research Method

### Questionnaire Design and Variable Measurement

In this paper, we used the questionnaire survey to obtain data and used SPSS25.0 to sort out the data and test the survey’s reliability and validity. After that, we applied the multiple regression model to analyze the data. The questionnaire had two parts: the user’s basic information and variable measurement.

The basic information part included gender, age, educational background, and users’ choice, which is the most commonly used transportation app. The scale design based on Likert Scale 5 combines the mature questionnaire and the actual needs of this study. In the variable measurement part, the items measuring the same variable are grouped into one group, consisting of 5 variables and 33 measure items. The questionnaire’s measurement indicators partly come from the maturity scale and partly are self-made indicators. The user activity is combined with the respondents’ willingness and behavior to use the app continuously. Service quality and system quality come from the D&M information system success model. The promotion effect is the extended variable based on the D&M information system success model. User preference is the extended variable too, which comes from the technology acceptance model. See Table 1 for variables’ detail.

**TABLE 1:** Variable connotation.

Variables	Connotations
SQ Service Quality	Preferential policies
	Comment channel
	Timely response and solution of problems
XQ System Quality	Easy to use
	Suitable for use
PE Promotion Effect	Browse
	Travel
	Comment
UP User Preference	User trust
	User interest
	User habit
UA User activity	Willingness to use
	Use behavior

### Data Collection

This questionnaire launched on February 26th, 2020, and ended on April 22nd, 2020. To improve the representativeness of the sample, we scientifically designed the sampling frame, used the multistage stratified sampling method, and tried to expand the sample size as more as possible. The multistage stratified sampling can be more scientific, representative, and efficient in inferring the whole population according to the sample population’s thoughts, behaviors, and cognition. Beijing has 16 administrative districts. In the sampling process, in the first stage, we divided each district into three regions according to its geographical location: the central area, the suburbs, and the outer suburbs. In the second stage, according to the economic conditions of each district, we selected one district with the best economy and one district with the worst economy in three regions (6 districts in total). In the third stage, we selected three communities in each district by simple random sampling (18 communities in total). In the fourth stage, we divided the samples into male and female groups and extracted independent random samples. We distributed 58 questionnaires in each community, and the ratio of male samples to female samples was 1 to 1.

We distributed a total of 1,044 questionnaires, each respondent filled out one questionnaire. We finally collected 935 questionnaires, with a recovery rate of 89.6%. After collecting the questionnaires, we filtered some questionnaires with lower quality through careful screening. The screening criteria are as follows:

Respondents must use the “Beijing One Card” app or the “Bus Code” app. If not, the questionnaire will be invalid.

The questionnaire sets two fake questions to test whether the respondents are thoughtfully answering the questions. If they choose the wrong answer, the data are invalid.

The questionnaire sets the answer limit. Respondents can only answer once. They need to log in to their private accounts when filling out the questionnaire.

Among the data we collected, 392 users selected the “Beijing One Card” app, 174 users liked the “Bus Code” app (the Beijing area), 301 users chose to use both two apps, and 68 invalid questionnaires. Because this paper is based on comparing two public transportation apps, the users who use both apps are not valid data. The basic data are 566 users who choose the “Beijing One Card” app or the “Bus Code” app (the Beijing area) alone. According to the meticulous degree of users’ answers, 344 questionnaires were selected for analysis after the strict screening. Among them, 243 users selected the “Beijing One Card” app, and 101 users chose the “Bus Code” app (the Beijing area). Table 2 shows the detail.

**TABLE 2:** A statistical table of the basic situation of respondents.

Variable	Category	Frequency	Percentage(%)
Gender	Male	153	44.5%
	Female	191	55.5%
	Total	344	
Favorite app	“Beijing One Card”	243	71%
	“Bus Code”	101	29%
	Total	344	
Age Range	Under 18 years old	8	2%
	19–35 years old	302	88%
	36–59 years old	27	8%
	After the age of 60	7	2%
	Total	344	
Educational Background	High school education degree and below	19	6%
	Bachelor’s degree	278	81%
	Master’s degree	35	10%
	Doctoral degree	12	3%
	Total	344	

### Analytical Method

We used SPSS25.0 to analyze data based on the multiple regression model. Before multiple regression analysis, we used the Pearson correlation test to test each variable and used the VIF variance inflation factor to judge whether there was multicollinearity of variables. Then, we used multiple regression analysis to summarize the main influencing factors of user activity. Finally, we compared two apps by comparative analysis.

## Analysis Results

We used SPSS 25.0 to test the reliability of questionnaire data. The Cronbach’s α values of service quality, system quality, promotion effect, user preference, and user activity are 0.775, 0.845, 0.834, 0.852, and 0.878, respectively. The overall Cronbach’s α coefficient of the questionnaire is 0.925, which is bigger than 0.7. It shows that the overall reliability of the questionnaire data is good and passes the test. Besides, the KMO value of the questionnaire is 0.907, which shows the questionnaire has good validity.

### Government-Provided App: “Beijing One Card”

Through the Pearson correlation test, Pearson correlation coefficients of service quality, system quality, promotion effect, and user preference with user activity are 0.389, 0.539, 0.490, and 0.561, which all pass the significance test with a significance level of 1%. Therefore, these four independent variables have a significant positive correlation with user activity. The multiple regression analysis results are shown in Table 3.

**TABLE 3:** Multiple regression results.

Model	Unstandardized coefficients		Standardized coefficients	*t*	Sig.	Collinearity statistics	
	B	Standard error	Trial version			Tolerance	VIF
(Constant)	0.238	0.341		0.698	0.486		
Service quality	–0.077	0.076	–0.072	–1.014	0.312	0.482	2.074
System quality	0.571	0.084	0.379	6.777	0.000	0.769	1.301
Promotion effect	0.144	0.072	0.184	2.011	0.045	0.286	3.494
User preference	0.325	0.104	0.287	3.127	0.002	0.285	3.514

*R^2^ = 0.428, Adjustment R^2^ = 0.418, F = 44.474 (P < 0.05).*

Table 3 shows that the maximum value of all variance inflation factors VIF (variance inflation) is 3.514, which meets the standard of 0∼10, so there is no multicollinearity of variables. The regression coefficients of system quality, promotion effect, and user preference have passed the significance test significantly different from zero (Sig < 0.05). The regression coefficient of system quality is 0.571, which indicates that system quality has a significant positive impact on user activity, and its impact coefficient is 0.571. The regression coefficient of the promotion effect is 0.144, which shows that the promotion effect has a significant positive impact on user activity, and its influence coefficient is 0.144. The regression coefficient of user preference is 0.325, which indicates that user preference has a significant positive influence on user activity, and its influence coefficient is 0.325. The regression coefficient of service quality did not pass the significance test (Sig > 0.05). Therefore, H2, H3, and H4 are confirmed in the “Beijing One Card” app, while H1: “the service quality has a significant positive impact on user activity” did not pass the test. Therefore, the multiple regression equation between service quality, system quality, promotion effect, user preference, and user activity can be summarized as follows:


(2) $$Y1=0.238+0.571^{*}XQ+0.144^{*}PE+0.325^{*}UP$$

The user activity of the “Beijing One Card” app is affected by system quality, promotion effect, and user preference. Service quality does not affect it. According to the impact coefficient rank, system quality is most significant; user preference and promotion effect are behind. System quality has a positive impact on user activity. The quality of the app system is directly related to user experience. The higher the system quality, the better the user experience. Only by making users feel satisfied will the user’s activity of this app be improved. Similarly, the lower the system quality and the worse the user experience, which virtually reduces the user’s satisfaction, the user becomes inactive. User preference also has a positive impact on the user’s activity. User preference is a psychological mechanism for users to integrate trust, interests, and habits. It plays an essential role in deciding which app to use, and it can also determine the user’s activity on the app. Although the promotion effect of the platform has a minor influence on user activity, it is still necessary. If the government does not promote this app, it is hard for users to know its existence.

In this study, the service quality does not affect the user activity of “Beijing One Card,” which conflicts with the views of most literature. Existing researchers (Chen et al., 2017, Chen Q. et al., 2020; Liu et al., 2017; Sun et al., 2017; Huang et al., 2020; Ming and Cao, 2020) believe that service quality has a positive and significant impact on user satisfaction, and user satisfaction has a positive and significant effect on users’ willingness to continue using. The low quality of service is the critical factor that users do not use continuously. If the quality of service affects users’ continuous use, it will also affect users’ activity. However, in this paper, we find that the service quality does not affect “Beijing One Card” user activity. The reason may be that there are few comparative studies based on the user activity of government-provided apps and business-provided apps in the existing literature. Still, these pieces of research only from the perspective of government-provided apps or business-provided apps carried out users’ willingness and behavior of continuous use. If they also consider comparing government-provided apps and business-provided apps, the results may be different.

### Business-Provided App: “Bus Code” (the Beijing Area)

Through the Pearson correlation test, Pearson correlation coefficients of service quality, system quality, promotion effect, and user preference with user activity are 0.223, 0.591, 0.216, and 0.257, which all pass the significance test with a significance level of 1%. Therefore, these four independent variables have a significant positive correlation with user activity. The regression analysis results are shown in Table 4.

**TABLE 4:** Multiple regression results.

Model	Unstandardized coefficients		Standardized coefficients	*t*	Sig.	Collinearity statistics	
	B	Standard error	Trial version			Tolerance	VIF
(Constant)	0.828	0.458		1.809	0.074		
Service quality	0.015	0.097	0.015	0.156	0.877	0.696	1.437
System quality	0.704	0.100	0.606	7.053	0.000	0.857	1.166
Promotion effect	0.180	0.081	0.255	2.229	0.028	0.484	2.067
User preference	–0.097	0.129	–0.089	–0.752	0.454	0.449	2.229

*R^2^ = 0.393, Adjustment R^2^ = 0.368, F = 15.554 (P < 0.05).*

Table 4 shows that the maximum value of all variance inflation factors VIF (variance inflation) is 2.229, which meets the standard of 0∼10, so there is no multicollinearity of variables. The regression coefficient of system quality and promotion effect passed the significance test. It was significantly different from zero (Sig < 0.05). The regression coefficient of system quality was 0.704, indicating that system quality had a significant positive impact on user activity. The impact coefficient is 0.704. The regression coefficient of the promotion effect is 0.180, which shows that the system quality has a significant positive impact on user activity. The impact coefficient is 0.180. The service quality and user preference regression coefficients failed to pass the significance test (Sig > 0.05). Therefore, in the business-provided apps “Bus Code” (the Beijing area), H2 and H3 passed the test, while H1: “Service quality has a significant positive impact on user activity” and H4: “User preference has a significant positive impact on user activity” did not pass the test. According to the above results, the multiple regression equation is:


(3) $$Y2=0.828+0.704^{*}XQ+0.180^{*}PE$$

In the business-provided app “Bus Code” (the Beijing area) user activity is affected by system quality and promotion effect. In contrast, service quality and user preference have no impact on it. This conflicts with previous research results. Most existing literature points out that service quality has a positive and significant effect on user satisfaction. User satisfaction has a positive and significant impact on users’ willingness to use continuously. So, service quality positively and significantly affects users’ willingness to use continuously (Chen et al., 2017; Chen Q. et al., 2020; Liu et al., 2017; Sun et al., 2017; Huang et al., 2020; Ming and Cao, 2020). Whether directly or indirectly, the quality of service should have affected users’ continuous use and directly affected users’ activity. However, in studying the influencing factors of user activity of the business-provided app “Bus Code” (the Beijing area), service quality does not affect user activity. Besides, the user preference does not affect the user activity of the business-provided app “Bus Code” (the Beijing area), too. This result is also contrary to previous research viewpoints. The perceived trust of external drivers impacts users’ continuous use behavior. Since trust is part of user preference, user preference should impact users’ activity. Zhong (2016), Ming and Cao (2020), and Zhang et al. (2020) also pointed out that habits have an impact on users’ continuous use behavior. Thus, user preference should also affect users’ activity. However, since they study from the perspective of a single subject (government-provided apps or business-provided apps), their results may not be general.

## Discussion

Through the comparison of multiple regression equations, the user activity of the government-provided app “Beijing One Card” is affected by system quality (0.571), promotion effect (0.144), and user preference (0.325). According to the influence coefficient, the system quality accounts for a large proportion. It shows that users who choose “Beijing One Card” have a significant factor: They favor the system quality of the app, choose to use it, and stay active because of its high system quality. The user activity of the business-provided app “Bus Code” (the Beijing area) is affected by system quality (0.704) and promotion effect (0.180). From the impact coefficient, we can see that the system quality coefficient of the app is also huge, which indicates that the users who choose the “Bus Code” (the Beijing area) and use it continuously remain active because of its high system quality too. This result is in line with the Guo and Ming’s (2020) and Pan’s (2020) opinion, they found that system quality has a significant impact on the public’s willingness to use continuously and user activity. The system quality coefficient of the business-provided app “Bus Code” (the Beijing area) is higher than the government-provided app “Beijing One Card.” It shows that, compared with “Beijing One Card,” the provider of “Bus Code” pays more attention to the system quality and focuses on the system quality. This finding could contribute to the development of government apps. The government should pay attention to system quality when developing government apps. According to the latest achievement of modern quality management theory, “quality is equal to customer satisfaction” and the research findings of many scholars (system quality has a significant positive impact on customer satisfaction). This paper suggests that both government-provided apps and business-provided apps should practically apply the D&M information system success model to life and pay attention to the improvement of apps’ system quality.

The promotion effect coefficients of the government-provided app “Beijing One Card” (0.144) and the business-provided app “Bus Code” (the Beijing area) (0.180) are relatively small, and there is no significant difference between the two apps. Few users choose to use each app because of the platform’s promotion effect, and the app’s promotion effect has little influence on the user’s activity. This result is different from the existing literature’s suggestions on the apps. Wang and Ding (2019) suggest that apps should pay more attention to publicity and promotion to improve users’ continuous use and activity. However, this paper suggests that whether it is a government-provided app or a business-provided app, the focus of work should not be mainly on the promotion effect of the platform but should take “meeting the needs of users” as the starting point and foothold. They, also, should choose the strategy of “appropriate promotion and focus on quality.”

**FIGURE 3:**
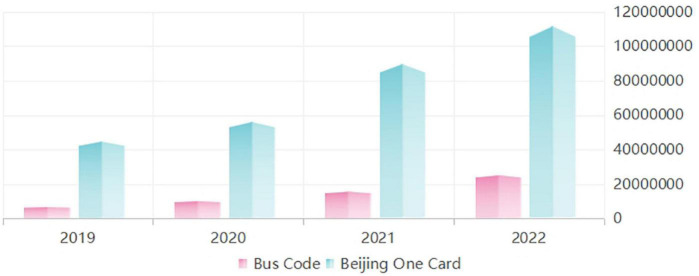
Total downloads of “Beijing One Card” and “Bus Code” from 2019 to 2022.

User preference is the most significant difference in influencing factors of user activity between the two apps. User preference affects the user activity of “Beijing One Card” but does not affect the user activity of “Bus Code” (Beijing area). It shows that their user preferences partly drive the users who choose to use the government-provided app “Beijing One Card.” In contrast, the users who prefer the business-provided app “Bus Code” (the Beijing area) pay more attention to the system quality and are mainly affected by the system quality, not user preferences. This finding of the “Beijing One Card” solves the user activity problem, solving the mystery of low user activity. It directly indicates that users prefer the government-provided app “Beijing One Card.” This result can also be proved by comparing the total downloads of the two apps. Users like to download the government-provided app “Beijing One Card.” See Figure 3 for details. As of May 9, 2022, the total number of downloads of the “Beijing One Card” app was 111,658,703 times. The total number of downloads of the “Bus Code” app (the Beijing area) was 25,195,452 times.

User preferences determine apps’ user activity. User preference focuses on users’ psychological tendencies, which shows users’ psychological identification with certain apps. The reason why users in Beijing prefer the government-provided app “Beijing One Card” is that they have a solid psychological identity with the government-provided apps. The technology acceptance process of government apps and business apps is different. Due to the authority and reliability of government-provided apps, such apps are easy for citizens to accept and continue to use. Given the public service attribute of the “Beijing One Card” app, the government itself has the unique, innate advantages of reliability, authority, and security; its construction concept of “people-centered” makes it easier to form users’ psychological identity. Because government apps have better citizen trust, they tend to have better citizen participation.

Government apps are different from other government platforms (websites, microblogs, etc.). The uniqueness of government apps is simple and efficient. Compared with government websites, government apps are mobile and can be used by users more conveniently. While government websites can also provide government services for citizens, the complexity of the process tends to discourage people. Government microblogging is a channel for the government to release official information. Compared with government microblogs, government apps can provide more comprehensive services. They can provide not only government information but also routine affairs. Government apps follow their own rules in technology adoption and innovation processes. These apps should not only exist for legal procedures, but also for the convenience of citizens.

In developing and promoting government-provided apps, the government provides high-quality transportation apps (*products*) on the network platform (*place*) that everyone can download. Based on the *4Ps theory*, they are combined with a flexible *price* adjustment, comprehensive preferential *policies*, and another strategy, which successfully guides users’ preferences. The functions of “Beijing One Card” and “Bus Code” are similar, providing public transportation services for citizens. However, the “Bus Code” user incentive measures are slightly inferior to “Beijing One Card.” “Beijing One Card” includes ordinary bus cards, student bus cards, short-term bus cards, Beijing-Tianjin-Wing exchange cards, and other card types, even offering discounts to students. The “Bus Code” is “treated equally.” The price is the same and does not have such a preferential policy.

Most scholars believe that factors such as service quality will affect users’ satisfaction, and satisfaction will affect continuous use (Chen et al., 2017; Chen Q. et al., 2020; Liu et al., 2017; Sun et al., 2017; Huang et al., 2020; Ming and Cao, 2020). The user’s continued use of the app has an equal relationship with the user activity. If the user cannot use this app continuously, the user’s activity research does not exist. According to previous viewpoints, service quality affects users’ continuous use, directly or indirectly affecting users’ activity. However, in this study, the service quality does not influence the government-provided app or business-provided app user activity. This result negates the views of most scholars and accords with the viewpoint of Liu and Wei (2020). They think that the quality level does not affect the users’ willingness to use continuously. Why does the service quality not affect the user activity of these two apps? It is because of the nature of these two apps. The “Beijing One Card” app and the “Bus Code” app are both public transportation apps. Users have low requirements for the service quality of these apps. Both apps have an online customer service center. If users have any questions, they can consult with the customer service staff. However, because the monetary amount involved in each trip is small (only about two RMB), users rarely need to consult questions with customer service staff. Besides, both apps offer transparent services, so the probability of disputes is low. Therefore, users pay little attention to whether the customer service can resolve their questions quickly, even if the conflict did exist. Customer service could not always offer a helpful solution. The timeliness and responsiveness of service quality cannot play a significant role in researching public transportation apps. This study speculates that users have higher requirements for the service quality of shopping apps because such apps involve commodity consultation and commodity return or exchange service. Although the service quality in the transportation app does not affect the user activity, it does not mean that the government and enterprises as providers can completely ignore the service quality. Service quality is the “reassurance” of app promotion. Combining the D&M IS success model and TAM, providers should fully consider the needs of users, provide comprehensive service, and solve complex problems in time to consolidate citizens’ trust, develop user preference, improve citizen participation, and consolidate the user activity in the app.

## Conclusion, Limitations, and Future Research

In this study, we examined three questions: What factors influence user activity of government-provided and business-provided apps? What are the differences between these two kinds of apps’ influencing factors? How to improve the user activity of apps? Although the findings of the previous studies have suggested that service quality is influencing user activity in apps, our study found service quality does not affect user activity in public transportation apps. System quality and promotion effect both affected government-provided app “Beijing One Card” and business-provided app “Bus Code.” User preference plays a critical role in distinguishing the influencing factors of user activity between the two kinds of apps. Few previous studies have focused on user preferences. This is an important finding in the understanding of the influence factors of apps’ user activity. Therefore, this result shows that to improve the user activity of apps, providers need to focus on user preference. In particular, the government should cultivate user preferences to consolidate citizen trust and improve citizen participation.

### Theoretical Contributions and Policy Implications

This study is the first to compare and analyze the influencing factors of government/business-provided public transportation apps’ user activity. It is interesting to find that user preference is the key factor that affects app user activity. This paper suggests that the app providers should make more outstanding efforts to cultivate user preference and improve the user activity of government-provided and business-provided apps.

Firstly, based on the principle of “improving the serviceability and service level of mobile government affairs,” the governments should focus on the system quality, ensure to provide higher service quality, continuously revise the government-provided apps, constantly improve and enhance the user experience, and solidify the user preference of the existing active users to consolidate citizen trust and improve citizen participation. Good system quality is a prerequisite for most users. If most users are active in this app, it will make the social proof cues and stimulate other users’ interest in continuous use. Social proof cues - its effectiveness is based on the psychological effect of social norms that are defined as “rules and standards that are understood by members of a group and that guide or constrain social behavior without the force of laws” (Cialdini and Trost, 1998) and that emerge from “interaction with others, they may or may not be stated explicitly, and any sanctions for deviating from them come from social networks, not the legal system” (Cialdini and Trost, 1998). Social orientation refers to watching others’ actions and deriving the meaning and implications for one’s own decision since others’ behavior is assumed to be based on sound reasoning. Apps’ user activity will be able to significantly increase by displaying a message indicating that the majority of citizens have high activity on these kinds of apps.

Secondly, through the feedback from users and market research, app providers can understand the needs of users, especially the needs of teenagers and the elderly, thereby optimizing the functions of the app system and catering to the user preference. Humans tend to pay attention to things readily at hand (Shirley and Lee, 2016). Moreover, human inertia makes them dislike complicated electronic programs. Since app suppliers cannot change human inertia, they should start from the app program. The app program should be as simple as possible to achieve the real purpose of convenience.

Thirdly, based on catering to user preference, it is also necessary to cultivate user preference. On the one hand, governments should increase publicity and education for young users to help them get familiar with the government-provided apps, consolidate users’ trust, cultivate users’ habits, and promote their user preferences. On the other hand, governments should pay attention to the silver-haired people and let the mobile government service cover more people, especially the elderly. The General Office of the State Council pointed out that “we should focus on high-frequency issues and service scenarios involving the elderly, adhere to the parallel of traditional service methods and intelligent service innovation and effectively solve the difficulties encountered by the elderly in using intelligent technology” (General Office of the State Council, 2020). From the aspects of transportation, daily medical treatment, daily consumption, cultural activities, and public services for the elderly, the governments and other providers should pay attention to all aspects of the life of elderly and solve the problem that the elderly are difficult to use mobile services. The service designs are needed to be convenient for the elderly, “promote the transformation of Internet application, make it also suitable for the elderly, and carry out intelligent technology education for the elderly” (General Office of the State Council, 2020), help them integrate into the new form of the service mode, and then cultivate the user preference of the elderly.

Furthermore, the government-provided apps can focus on and mine the user behavior data in the operation process of the app, and focus on the service object through the public policy to improve the decision-making efficiency and influence the user’s preference. After affecting user preferences, these measures can consolidate citizen trust. Many studies have shown that good citizen trust can promote citizen participation.

Finally, to promote citizen participation in the development of China’s e-government, this paper argues that practice should be from the following points: When the “digital divide” between different regions and classes cannot be effectively bridged in the short term, governments at all levels should, while actively developing e-government, retain and open some traditional forms and channels of government affairs that are convenient for citizens in underdeveloped regions and other vulnerable citizens to participate in politics and express their willingness to guarantee the political participation rights of each legitimate citizen as fair as possible, and prevent citizens without e-government information network resources from being excluded or “squeezed out” by large and pure e-government models. In addition, to further strengthen the construction of newspapers, radio, television, and other traditional information media investment, to ensure that vulnerable citizens’ right to know government policy, to a certain extent, is conducive to making up for the huge “digital divide”; improve the implementation of the legal system of citizen participation in e-government to provide an institutional guarantee for citizen participation. While high levels of citizen political participation ultimately depend on a variety of factors, institutional guarantees are most important, including safeguarding citizens’ rights to policy information, participation, and oversight. Safeguard smooth channels of citizen participation. By establishing a complete set of rules and regulations, the content, methods, and ways of citizens’ participation in e-government are stipulated so that citizens can achieve regular and institutionalized political participation according to legal procedures.

These five aspects are connected with theories of technology acceptance and D&M information system success in government. At the same time, these suggestions make up for the shortcomings of existing literature and enrich the existing theoretical basis.

### Limitations

Compared with the “Bus Code” (the Beijing area) of the business-provided app in the field of transportation, the government-provided app “Beijing One Card” is more preferred by users, which makes it have higher download volume and higher user activity. This research is just a basic research, and there are still many shortcomings. The basic proposed model still needs to be improved.

Firstly, the shortcomings of data collection methods. Variable collection depends on self-assessment questionnaire, the subjective report data may exist homologous deviation, and they also cannot eliminate social acceptability. This study mainly through the investigation of the design program (such as the use of two time points measurement, anonymous processing, model test) to solve the above problems, which cannot be completely avoided. In addition, although this paper solves the problem of self-selection bias by reducing the difference between groups, it cannot be completely avoided. Sample representativeness cannot be fully fitted, only through multistage stratified sampling method to maximize its degree of the fitting.

Secondly, the shortcoming of the basic model. This basic model does not have the control variables, such as age and income. Users of different ages may have different user activities. Theoretically, middle-aged people are more active in government-provided apps because they often need to pay and do things through government-provided apps in daily life. Teenagers are relatively less active in government-provided apps, and they seldom use them except for education services and transportation services. The elderly should be the least active in government-provided apps because they are not used to new technology and always feel that they have difficulty using it. They do not easily adapt to the convenience provided by e-government services. And their children usually handle various procedures for them, so they rarely contact e-government apps. Moreover, the educational level can be increased in the model as the control variable. Users with different educational backgrounds may have different user experience. Theoretically, users with higher educational levels are more likely to respond to e-government service policy, making the higher the user activity in government-provided apps. Besides, some remote areas have not yet connected to the Internet. Few people pay attention to e-government services.

Finally, user preference influences people’s s behavior by changing the decision-making process. However, user preference is not stable, and there may be heterogeneity among different populations. Compared with the initial research group, a certain user preference may not be so common in a specific target group. In addition, the intensity of user preference may not be as obvious as expected. Furthermore, a certain user preference may interact with other rules, resulting in spillover effects, thereby offsetting the expected effect (higher user activity).

### Future Research

This study found the key factor affecting app user activity: user preference. This result inspires both government app providers and business app providers. Providers should pay attention to user preferences if they want to improve the user activity of an app. In addition, this result has implications for the government that are different from enterprises. People’s preferences for government apps come mainly from their trust in the government. The government should use its authoritative and reliable characteristics to improve better services for citizens, establish a good government image, and improve the credibility of the government to improve citizen participation. In future research, the following aspects can be carried out:

Scholars can explore the deep integration of government apps and user preferences, focusing on the analysis of user activity of government apps, which is different from citizen adoption and citizen satisfaction. The complementary relationship between the two perspectives determines the inevitable trend of their mutual integration. Therefore, in the future, it is necessary to further explore the interactive relationship between user preference and individual behavior characteristics and the overall mechanism of its influence on the activeness of government app users to form a multidimensional understanding of the influencing factors and mechanism of government app user activity. Based on clarifying the relevant concepts and identifying the influencing factors and mechanism of user activity of different types of government apps, from the perspective of the complementarity of user preference and user activity theory, from the single influence to the common influence, the logical framework of the driving mechanism that limits the user activity of citizen-government apps from the dual perspective of “user activity – user preference” is constructed. At the same time, special attention is paid to the analysis of the interaction between different intervention strategies and the spillover and superposition effects between different policy instruments.

Furthermore, scholars can promote the wider and deeper application of user preferences in government app research. Existing research, mostly through technology acceptance model TAM, technology acceptance extension model TAM2, technology acceptance and use of integration theory UTAUT, innovation diffusion theory DOI to study e-government citizen satisfaction, citizen adoption and other research topics. However, these studies mostly occur in the policy context where citizens have a greater right to independent choice. For policy practices aimed at restricting citizens’ specific freedom of behavior, there are few related researches, which makes it impossible for scholars to fully tap the great potential of behavioral science knowledge to improve the effectiveness of public policies. Therefore, future research should strive to apply behavioral science to a wider range of e-government policy areas, especially focusing on individual behaviors with negative externalities, and promoting relevant academic accumulation. At the same time, scholars should fully tap the potential of behavioral economics and behavioral science in expanding the government policy toolbox. Scholars should transcend the policy design thinking that only uses individual cognitive errors and strive to explore the deep-seated reasons behind the active users of government apps.

## Data Availability Statement

The original contributions presented in the study are included in the article/supplementary material, further inquiries can be directed to the corresponding author/s.

## Author Contributions

YG provide substantial contributions to the conception or design of the work and the acquisition, analysis, interpretation of data for the work, drafting the work or revising it critically for important intellectual content, gave the final approval of the version to be published, and agreement to be accountable for all aspects of the work in ensuring that questions related.

## Conflict of Interest

The author declares that the research was conducted in the absence of any commercial or financial relationships that could be construed as a potential conflict of interest.

## Publisher’s Note

All claims expressed in this article are solely those of the authors and do not necessarily represent those of their affiliated organizations, or those of the publisher, the editors and the reviewers. Any product that may be evaluated in this article, or claim that may be made by its manufacturer, is not guaranteed or endorsed by the publisher.
