# Corrigendum: Does user preference matter? A comparative study on influencing factors of user activity between government-provided and business-provided apps

**DOI:** 10.3389/fpsyg.2022.986670

**Published:** 2022-08-03

**Authors:** Yuanyuan Guo

**Affiliations:** Department of Government Management, Beijing Normal University, Beijing, China

**Keywords:** government-provided app, business-provided app, digital government, user behavior, user preference

In the published article, the year that the questionnaire took place was incorrectly stated.

A correction has been made to **Research Method**, “*Data Collection*,” Paragraph 1. The corrected paragraph is shown below.

“This questionnaire launched on February 26th, 2020, and ended on April 22nd, 2020. To improve the representativeness of the sample, we scientifically designed the sampling frame, used the multistage stratified sampling method, and tried to expand the sample size as more as possible. The multistage stratified sampling can be more scientific, representative, and efficient in inferring the whole population according to the sample population's thoughts, behaviors, and cognition. Beijing has 16 administrative districts. In the sampling process, in the first stage, we divided each district into three regions according to its geographical location: the central area, the suburbs, and the outer suburbs. In the second stage, according to the economic conditions of each district, we selected one district with the best economy and one district with the worst economy in three regions (6 districts in total). In the third stage, we selected three communities in each district by simple random sampling (18 communities in total). In the fourth stage, we divided the samples into male and female groups and extracted independent random samples. We distributed 58 questionnaires in each community, and the ratio of male samples to female samples was 1 to 1.”

In the published article, there was also an error in the stated KMO value.

A correction has been made to **Analysis Results**, Paragraph 1 and paragraph 2. The corrected paragraph is shown below.

“We used SPSS 25.0 to test the reliability of questionnaire data. The Cronbach's α values of service quality, system quality, promotion effect, user preference, and user activity are 0.775, 0.845, 0.834, 0.852, and 0.878, respectively. The overall Cronbach's α coefficient of the questionnaire is 0.925, which is bigger than 0.7. It shows that the overall reliability of the questionnaire data is good and passes the test. Besides, the KMO value of the questionnaire is 0.907, which shows the questionnaire has good validity.”

The authors apologize for these errors and state that they do not change the scientific conclusions of the article in any way. The original article has been updated.

## Publisher's note

All claims expressed in this article are solely those of the authors and do not necessarily represent those of their affiliated organizations, or those of the publisher, the editors and the reviewers. Any product that may be evaluated in this article, or claim that may be made by its manufacturer, is not guaranteed or endorsed by the publisher.

